# Wastewater Surveillance of SARS-CoV-2 in Slovenia: Key Public Health Tool in Endemic Time of COVID-19

**DOI:** 10.3390/microorganisms12112174

**Published:** 2024-10-29

**Authors:** Natalija Kranjec, Andrej Steyer, Tjaša Cerar Kišek, Tom Koritnik, Tea Janko, Maja Bolješić, Vid Vedlin, Verica Mioč, Barbara Lasecky, Tatjana Jurša, José Gonçalves, Herbert Oberacher, Alenka Trop Skaza, Mario Fafangel, An Galičič

**Affiliations:** 1 National Institute of Public Health, Trubarjeva ulica 2, 1000 Ljubljana, Slovenia; 2 National Laboratory for Health, Environment and Food, Prvomajska ulica 1, 2000 Maribor, Slovenia; 3 Marine and Environmental Sciences Centre, Aquatic Research Network Associate Laboratory, NOVA School of Science and Technology, NOVA University Lisbon, 2829-516 Caparica, Portugal; 4 Institute of Legal Medicine and Core Facility Metabolomics, Medical University of Innsbruck, Muellerstrasse 44, 6020 Innsbruck, Austria

**Keywords:** wastewater-based epidemiology, wastewater-based surveillance, sewage, COVID-19, SARS-CoV-2

## Abstract

With the reclassification of COVID-19 as an endemic disease and the relaxation of measures, Slovenia needed a complementary system for monitoring SARS-CoV-2 infections. This article provides an overview of the epidemiological situation of SARS-CoV-2 in Slovenia using a wastewater surveillance system, demonstrating its usefulness as a complementary tool in epidemiological surveillance. This study found that estimated SARS-CoV-2 infections in Slovenia peaked in September 2022 and showed a declining trend with subsequent lower peaks in March–April and December 2023, mirroring the trends observed from clinical data. Based on both surveillance systems, the most prevalent variant in 2022 was BA.5. By 2023, BQ.1 and other Omicron variants increased in prevalence. By the end of 2023, XBB sublineages and the BA.2.86 variant had become predominant, demonstrating consistent dynamic shifts in variant distribution across both monitoring methods. This study found that wastewater surveillance at wastewater treatment plants in Slovenia effectively tracked SARS-CoV-2 infection trends, showing a moderate to strong correlation with clinical data and providing early indications of changes in infection trends and variant emergence. Despite limitations during periods of low virus concentration, the system proved significant in providing early warnings of infection trends and variant emergence, thus enhancing public health response capabilities.

## 1. Introduction

At the beginning of 2022, European Union European Economic Area countries initiated a transition from widespread testing during the acute phase to more targeted and diagnostic-focused testing strategies during the post-acute phase of the SARS-CoV-2 pandemic. This shift was characterized by the continued emphasis on clinical surveillance and sentinel sampling as essential components of ongoing monitoring, all while optimizing the use of resources [1]. Slovenia followed a similar approach, by abolishing quarantine measures and free rapid antigen test screening at the end of February 2022 [2].

Following 1 April 2023, COVID-19 was reclassified into the category of endemic respiratory disease, meaning that the virus was expected to continue circulating in the population but at lower and more manageable levels, with less severe outcomes [3]. Reclassification has involved changes in public health measures such as testing protocols and the abolishment of isolation measures. Healthcare institutions are no longer required to provide testing facilities for the detection of SARS-CoV-2 infections, which previously had to be done within 24 h of ordering. Concurrently, the Ministry of Health in Slovenia has also ceased the daily surveillance of hospital bed capacities at standard and intensive care units [3].

**Figure 1:**
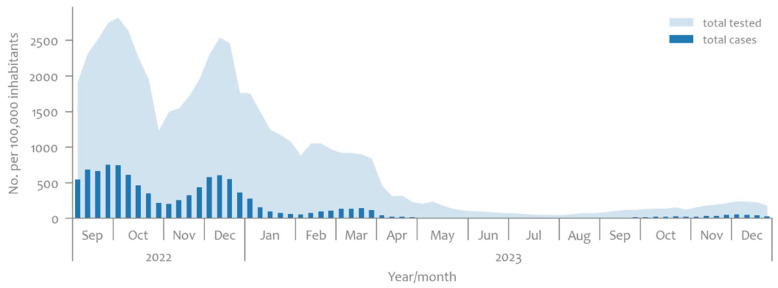
Timeline of weekly number of clinically tested individuals and positive cases per 100,000 inhabitants, 5 September 2022–31 December 2023, Slovenia [4].

With the above-mentioned mitigation of measures, the number of tests and identified cases of SARS-CoV-2 infections decreased, as seen in Figure 1 [4].

**Figure 2:**
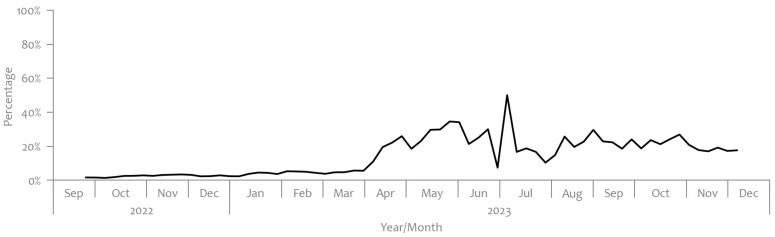
Percentage of samples from clinical cases that underwent sequence analysis [4].

The national strategy for the sequencing surveillance of SARS-CoV-2 variants in Slovenia focused on the early detection of variants with increased transmissibility or severity. It aimed to sequence at least 5–10% of PCR-positive samples [5]. As seen in Figure 2, between September 2022 and August 2023, the proportion of sequenced samples relative to the total weekly cases rarely surpassed 5%. However, there were specific months when this percentage exceeded that threshold; in January 2023, it reached a maximum of 14%, in June, it peaked at 12%, and in August, it reached 15%. Still, it should be noted that after April 2023, there was a significant decline in the number of individuals tested, and notably, the number of positive cases dropped from approximately 132 per 100,000 to a maximum of 10 per 100,000 inhabitants, as observed in Figure 1 [4]. This has made human testing data less reliable for epidemiological surveillance at the population level.

A supplementary SARS-CoV-2 surveillance system needed to be established to enable the monitoring of timely and representative trends of SARS-CoV-2 infection rates and the prevalence of SARS-CoV-2 variants with transitions to endemic disease with reduced clinical testing, utilizing non-invasive methods for individuals. This capability was recognized as vital for a comprehensive understanding of the virus’s dynamics within the population. A better understanding of the epidemic can also be achieved by monitoring the abundance of new virus variants. SARS-CoV-2 variants can have diverse impacts on various aspects of the virus’s behavior and its interactions with humans. These effects include changes in transmissibility, the severity of illness it causes, immune evasion (ability to evade the immune response from previous infections or vaccinations), and potential impacts on available treatments [6]. With this objective in view, Slovenia has started to establish an epidemiological surveillance system for SARS-CoV-2 in wastewater. The surveillance was piloted from September 2022 to January 2023 and continues to serve as a complementary surveillance system.

The aim of this article is to provide an overview of the epidemiological situation within the wastewater surveillance system for the SARS-CoV-2 virus in Slovenia. In this way, the usefulness of wastewater surveillance as a complementary tool for monitoring and managing SARS-CoV-2 infections is demonstrated.

## 2. Materials and Methods

### 2.1. Sampling Sites

Slovenia is a Central European country that is bordered by Austria, Hungary, Italy, and Croatia. Its population is about 2.1 million people. Slovenia is divided into 12 statistical regions, which play a crucial role in supporting regional development, aiding in professional planning, assessing the impact of regional policies, and conducting socio-economic analyses [7]. In Slovenia, urban wastewater is treated in 246 wastewater treatment plants (WWTPs) with environmental permits before it is discharged [8].

**Figure 3:**
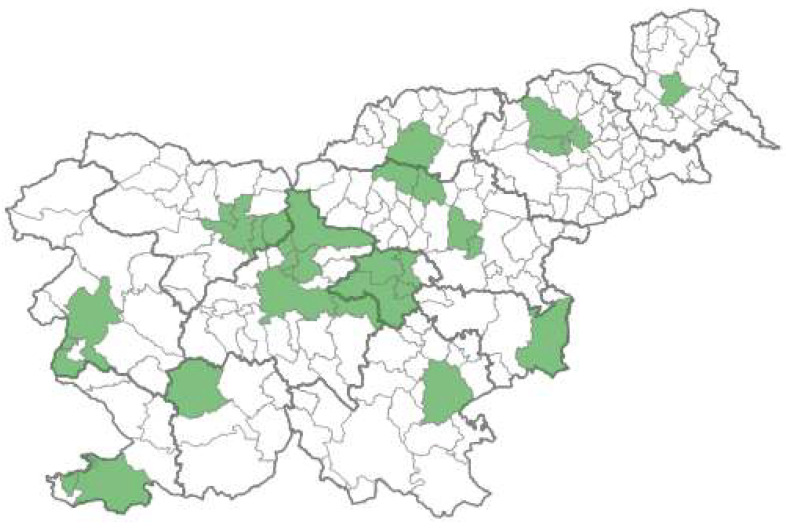
Catchment areas of the wastewater surveillance in Slovenia (green color).

The wastewater monitoring network covered 16 of the 246 Slovenian WWTPs. They receive wastewater from 33 municipalities in Slovenia with 730,887 inhabitants (34.2% of the total population) [9]. Surveillance of SARS-CoV-2 in wastewater is conducted in all statistical regions in the Republic of Slovenia (Figure 3). A list of the involved WWTPs, involved municipalities, and the proportion of the population of the statistical region connected to the monitored WWTPs is shown in Supplementary Table S1.

### 2.2. Wastewater Sampling and Transport

Influent wastewater samples were collected as 24 h composite samples using cooled autosamplers and transported to the laboratory at 4 °C within 24 h from the time of collection. The daily flow rates and temperature of wastewater were measured by the WWTP operator. Wastewater samples from each WWTP were used for microbiological and chemical analysis.

### 2.3. Sample Concentration

Upon arrival in the microbiological laboratory, samples were stored at 4 °C and processed within 24 h of arrival. The samples were thoroughly mixed, and 100 mL aliquots were taken for further analysis. All the wastewater samples were processed in a microbiological laboratory directly without heat inactivation in a biosafety cabinet. The equine arteritis virus (EAV) (0.1 mL of 4.5 × 107 TCID50/mL) was spiked into 100 mL of wastewater as a surrogate to monitor virus recovery. The EAV was grown in-house on the RK13 cell line (CCL-37, ATCC, Manassas, VA, USA), and its concentration was measured by the TCID50 method to determine the infectious unit per mL of the stock. Additionally, the number of EAV viral copies was quantified with qPCR in each batch. After spike-in, samples were mixed thoroughly and centrifuged at 3200× *g* for 30 min at 4 °C to settle the larger particles. The supernatant was concentrated at 3200× *g* at 4 °C for 20 min for each addition using Centricon^®^ Plus-70 10 kDa Centrifugal Filter Units (Merck KGaA, Darmstadt, Germany). The time of centrifugation was prolonged until a sufficient volume of the sample passed through the filter to reach a final concentrate volume of 0.5–1.5 mL. The concentrate was collected by centrifuging at 1000× *g* for 2 min and transferred to a new tube. The sample was stored at −80 °C until processing.

### 2.4. Nucleic Acid Extraction

Nucleic acids were extracted from the concentrates using the MagMaxx Wastewater Ultra Nucleic Acid Isolation Kit (Thermo Fisher Scientific Inc., Waltham, MA, USA) with the KingFisher Flex automatic extraction system (Thermo Fisher Scientific Inc., Waltham, MA, USA). The extraction was accomplished according to the manufacturer’s instructions with an initial sample volume of 200 µL and an elution volume of 100 µL. For every extraction, a negative control (PCR-grade water) was used.

### 2.5. Chemical Analysis

Samples underwent chemical analysis to determine the levels of chemical oxygen demand (COD). COD values were used for the estimation of the individual WWTPs catchment population, as described in the section “Wastewater-Based Epidemiological Indicators for SARS-CoV-2”.

Laboratory chemical analysis was carried out using validated and accredited standard methods in accordance with the requirements of SIST EN ISO/IEC 17025 [10]. Samples were stored in a laboratory at (5 ± 3) °C until individual analyses. The pH of the water samples was measured according to the SIST EN ISO 10523 standard [11] within 24 h from the time of collection. The samples were first filtered through a glass fiber filter using a vacuum filtration system, then dried at 105 °C and subjected to gravimetric analysis.

The COD was performed according to the SIST ISO 15705 standard [12] using pre-prepared commercial tests (NANOCOLOR tube tests, Macherey-Nagel GmbH & Co. KG, Germany). A total of 2 mL of the water sample was added to the pre-prepared test tube, mixed, and left for decomposition process at 150 °C for 120 min. After decomposition, the samples were cooled to room temperature and analyzed by spectrophotometry.

### 2.6. Quantitative Reverse Transcription Real-Time Polymerase Chain Reaction

We conducted quantification of SARS-CoV-2 and pepper mild mottle virus (PMMoV) by amplifying a 71 bp segment of the SARS-CoV-2 nucleocapsid gene and a 68 bp segment of the PMMoV replicase gene, respectively.

An RT-qPCR mix contained 9.5 µL PCR-grade water, 5 µL TaqMan Fast Virus 1-Step Master Mix (Thermo Fisher Scientific Inc., Waltham, MA, USA), 0.5 µL 20 µM primer solutions, 0.2 µL 20 µM probe solutions, and 5 µL of nucleic acid extract. A list of the utilized primers and probes can be found in Supplementary Table S2.

For quantification of EAV, the LightMix^®^ Modular EAV RNA Extraction Control (580) kit (Roche, Mannheim, Germany) was employed following the manufacturer’s instructions.

Amplification for all targets was carried out using a Quantstudio 5 device (Thermo Fisher Scientific, Waltham, MA, USA) under the following conditions: a reverse transcription (RT) step at 50 °C for 5 min, initial denaturation at 95 °C for 20 s, followed by 45 cycles of denaturation at 95 °C for 3 s, and extension at 60 °C for 30 s. Fluorescence thresholds were set to a value of 0.15 and the threshold of positive detection was set at <40 quantitative cycles. All samples, both undiluted and diluted at a ratio of 1:10, along with standards and negative controls (extraction and PCR), were tested in duplicates. The difference of at least 2.5 Ct value between the sample and its 10-fold serial dilution indicates that there was no significant inhibition and thus, the undiluted Ct value was used for further calculations. If the difference was less than 2.5 Ct values, the value of the diluted sample was used for further calculations and the formula was adjusted to include the extra dilution.

Standard curves were developed using the 2019-nCoV_N_Positive Control (IDT, Coralville, IO, USA), EAV RNA extraction control (TIB MolBIol, Berlin, Germany), and PMMoV in vitro RNA standard (TIB MolBIol, Berlin, Germany) at concentrations ranging from 106 to 101 gene copies per reaction. Standard curve calibration was included in every RT-qPCR run. Standard curve parameters and limit of detection for qPCRs are listed in Supplementary Table S3.

The SARS-CoV-2 and PMMoV concentrations in wastewater were calculated as described previously by [13] with Equation (1).

(1) $$RNA\;copy\;number\left(\frac{copy}{L}\right)=RNA\;copy\;number\left(\frac{copy}{reaction}\right)\times \frac{V\left(extracted\;DNA\right)}{V\left(RNA\;in\;each\;PCR\;reaction\right)}\times \;\frac{V\left(wastewater\;concentrate\right)}{V\left(wastewater\;concentrate\;for\;RNA\;extraction\right)}\times \frac{1000}{V\left(initial\;wastewater\right)}$$

To determine the recovery rate, the number of EAV genome copies recovered from the respective sample was divided by the number of seeded copies. The concentrations of SARS-CoV-2 and PMMoV were adjusted using the recovery rate of the respective sample. This involved dividing the calculated RNA copy numbers by the recovery rate.

### 2.7. Amplicon Sequencing and Analysis

A volume of 20 μL of the extracted nucleic acids was used for sequencing with Oxford Nanopore GRIDIon device (Oxford Nanopore Tech, Oxford, UK). First, RT was performed using Lunascript RT Supermix (New England Biolabs, Ipswich, MA, USA) by adding 5 µL of the RT mix to each 20 µL sample with the following PCR conditions: 25 °C for 2 min, 55 °C for 20 min and 95 °C for 1 min. RT was followed by amplicon construction. Two sets of primer pairs were used for amplicon generation: ARTIC V4.1 (IDT) and VarSkip V2 (New England Biolabs, Ipswich, MA, USA) to maximize possible amplicon generation despite possible mutations and amplicon fall-out. Each sample had 4 pools of amplicons (2 for ARTIC and 2 for Varskip). The reaction mixture was prepared with the following reagents: 12.5 µL Q5 Hot Start High-Fidelity 2X Master Mix, 3.7 µL specific primer pool, and 3.8 µL PCR-grade water. A total of 5 µL of the RT sample was added to each pool. The PCR conditions were as follows: 98 °C for 30 s, 35 cycles of 98 °C for 15 s, and 65 °C for 5 min).

Library construction was done using the ligation-based PCR-tiling protocol developed by Quick [14]. The samples were first end-prepped, and barcodes were ligated followed by adapter ligation. The libraries were then loaded on R.9.4 flow cells and a sequencing run was set to run for 12 h on a GRIDIon device. Basecalling was performed by the MinKnow software (v. 23.11.4) natively on the GRIDIon. As 2 sequences were generated for each sample (ARTIC and Varskip primer scheme), each was processed using the Wf-artic bioinformatic workflow (available on the webpage https://github.com/epi2me-labs/wf-artic (accessed on 1 December 2023). The BAM outputs for each sample were then combined and Freyja (available on the webpage https://github.com/andersen-lab/Freyja (accessed on 1 December 2023) was used for demixing and visualization. The tsv file produced by Freyja was used to generate the visualization on the SARS-CoV-2 dashboard [15]. Samples went through manual inspection to ensure the quality parameters were of sufficient value. For the quality, the –Q parameters in the Freyja tool as first set to 20, and using an in-house pipeline it was lowered to a minimum of 17, for the samples that did not pass a higher quality score. The resulting tsv file was then inspected to ensure samples used for reporting had a minimum coverage value of 35. Samples below this threshold were removed from the reporting table and were not used for visualization of the data.

### 2.8. Wastewater-Based Epidemiological Indicators for SARS-CoV-2

The epidemiological wastewater surveillance system utilized two key wastewater-based indicators: the estimated incidence rate of SARS-CoV-2 infections per 100,000 individuals within the catchment population, and the prevalence of specific SARS-CoV-2 variants.

The estimated number of SARS-CoV-2 infections per 100,000 catchment population was calculated by first calculating the normalized viral loads. Normalized viral loads were calculated by multiplying virus concentration with WWTP flow rate (L/day) and dividing the result by the equivalent population. WWTP’s equivalent population was defined by dividing COD concentrations in the 24 h sample with the per capita equivalents (120 g/day/person) [16,17]. The estimated number of infections was subsequently calculated by applying a conversion factor to the normalized viral loads. The conversion factor for each individual WWTP was determined by fitting the normalized viral loads to the number of active infections as identified from the surveillance system of human sampling with linear regression. We used the highest testing rate in the study period for this, between 5 September 2022 and 31 January 2023.

Prior to conducting the regression analysis, the data on measured normalized viral loads and active infections underwent essential pre-processing steps to ensure their suitability for modeling:

Logarithmic Transformation: To stabilize the variance and address skewness in the viral load data and active clinical cases, a logarithmic transformation was applied to each individually. This transformation involved taking the natural logarithm of each viral load value and number of active cases.
Outlier Removal: Outliers can significantly affect the results of linear regression models. Therefore, we performed outlier detection using Cook’s distance. A Cook’s distance greater than 1 or 4/n (where n is the number of data points) was used to determine outliers. All identified outliers were removed from further data analysis.

The data on the estimated number of SARS-CoV-2 infections per 100,000 catchment population from individual WWTPs were aggregated to assess the epidemiological situation across the entire country.

#### 2.8.1. Validation Procedure

The cross-correlation analysis served as a validation method to assess the accuracy and reliability of the estimated number of SARS-CoV-2 infections per 100,000 catchment population by comparing them with clinical data. The strength of the correlation was assessed according to the recommendations by [18]. In this process, temporal alignment was executed to account for the delays in the transmission, onset of symptoms, and reporting procedures between the two datasets. This validation process focused on the period following the determination of the conversion factor (1 February 2023, to 31 December 2023).

#### 2.8.2. Abundance of SARS-CoV-2 Variants from Wastewater Samples

The indicator presents the abundance of each SARS-CoV-2 variant in a wastewater sample at the inflow of the WWTP. It is based on the identification of specific mutations in the RNA genome of SARS-CoV-2 for each variant. The method of identification by sequencing is explained in the section “Amplicon Sequencing and Analysis”.

Data on the abundance of SARS-CoV-2 variants from individual WWTPs were aggregated to evaluate the epidemiological situation for the entire country.

### 2.9. Clinical Data

We collected clinical data on reported SARS-CoV-2 infections from the Slovenian Register of Communicable Diseases-NIJZ-48 [4]. This register provided detailed records from the national clinical surveillance system, which we used to analyze infection trends by calculating the number of active infections per 100,000 inhabitants. For the calculation of active infections, we considered an average infectious period for SARS-CoV-2, typically estimated to be around 10 days [19]. Additionally, we collected data on the prevalence of virus variants from the register, using whole-genome sequencing of clinical PCR samples, allowing us to monitor the abundance of different variants over time. These metrics served as complementary and comparable measures to the wastewater-based epidemiology data, enriching our comprehensive, multi-faceted assessment of the epidemiological situation.

### 2.10. Dissemination Strategy

Wastewater-based epidemiological indicators for SARS-CoV-2, as well as indicators derived from clinical data on active cases, were utilized for data visualization in the form of a dashboard on the Slovenian wastewater monitoring website for the purpose of dissemination. Developed using R and Rmarkdown, both the website and the indicators are tailored to meet the needs of a diverse audience, including the general public, experts, and authorities. The website can be accessed at the following link: https://modeliranje.nijz.si/epivode/epivode-c19.html (accessed on 24 February 2024) and is available in the Slovenian language. Additionally, our dissemination strategy specifically targeted healthcare providers and nursing homes. They received updates on the current COVID-19 epidemiological situation from the wastewater surveillance through the weekly public health risk assessment report from the National Institute of Public Health’s Emergency Operations Centre.

## 3. Results

**Figure 4:**
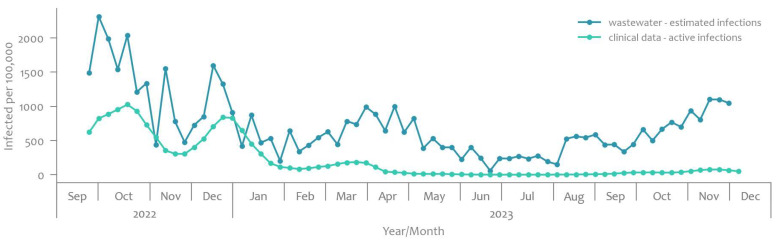
Time-series plot of the estimated number of SARS-CoV-2 infections per 100,000 inhabitants in Slovenia and the number of active clinically confirmed SARS-CoV-2 infections per 100,000 people in the catchment population.

Temporal trends in the estimated number of SARS-CoV-2 infections per 100,000 inhabitants based on the wastewater estimates and the number of active clinically confirmed SARS-CoV-2 infections per 100,000 inhabitants in Slovenia. As seen in Figure 4, the estimated infections displayed a varied trend over the 16-month surveillance period, with the highest number recorded in September 2022 (2312.3 per 100,000 inhabitants) and the lowest in June 2023 (60.8 per 100,000 inhabitants). Initially, the estimated infections peaked in September 2022 and gradually declined until the end of January 2023. Subsequently, the trend started to increase in February 2023, peaking again in March and April 2023. The estimated infections fluctuated thereafter, with another peak at the end of the year 2023. Peaks in the estimated number of infections that followed the first one in September 2022 were 2.3 times lower in March–April 2023 and 2.1 times lower in December 2023 than the one in September 2022.

The highest number of active infections as determined from human testing, was observed in October 2022 (1026.8 per 100,000 inhabitants), while the lowest was recorded in July 2023 (1.1 per 100,000 inhabitants). Initially, the active infections peaked in October 2022 and gradually declined until January 2023, reaching the lowest point. The trend started to increase in February 2023, peaking again in April and June 2023. The active infections fluctuated thereafter, with another peak observed in November 2023. The epidemiological situation for each individual WWTP is displayed in Supplementary Figure S1.

The validation process using cross-correlation showed the best fit at lag 1 (1 week). Out of 16 WWTPs, 5 exhibited a strong correlation between estimated infections and active clinically confirmed infections, while others exhibited a moderate correlation Table 1.

### Abundance of Mutations Specific to SARS-CoV-2 Variants

**Figure 5:**
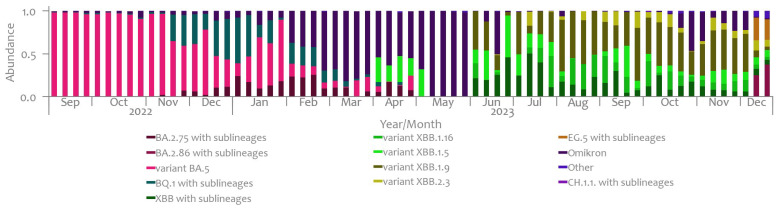
Abundance of SARS-CoV-2 variants, as determined based on the wastewater data, Slovenia, September 2022–December 2023.

For the observed monitoring period, the abundance of SARS-CoV-2 variants, as determined based on the wastewater data, is shown in Figure 5.

Based on the data collected from the wastewater samples in Slovenia, the BA.5 variant emerged as the dominant strain in September 2022, reaching up to 98%. The BQ.1 variant also appeared but in lower abundance. In November, the dominance of the BA.5 variant started to gradually decrease at the expense of the BQ.1, which peaked at 31% in week 47. In December 2022, the distribution further changed, with the BA.5 and BQ.1 accounting for 55% and 35%, respectively, by week 49. The BA.2.75 variant with sublineages was prevalent in less than 15% by the end of 2022 and peaked at 26% at week 8 in February 2023.

At the beginning of 2023, the dominance of the BA.5 and BQ.1 variants in the wastewater samples continued to decrease, and by the end of February, they accounted together for less than 25%. Between February and May 2023, the determination of the virus variant was predominately limited to the level of Omicron parent lineage, except for a few weeks in April where the XBB.1.5 lineage could be determined (at around 30%). Between June and the first half of December 2023, the predominant variants were XBB with the sublineages XBB.1.16, XBB.1.5, XBB.1.9, and XBB.2.3. In the second half of December 2023 variants BA.2.86 and EG.5 appeared, reaching 37% and 26%, respectively.

**Figure 6:**
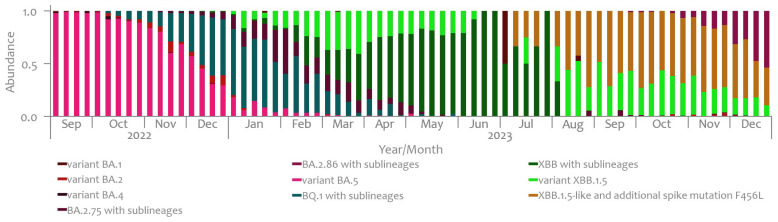
Timeline of SARS-CoV-2 variant abundances in clinical cases, Slovenia, September 2022–December 2023 [4].

During the observed period, a total of 22,825 clinical samples (10.07% of all infections in that period) were sequenced using whole-genome sequencing methods [4]. The abundance of SARS-CoV-2 variants, as determined based on the data from clinical cases, is shown in Figure 6.

From September 2022 to early December of the same year, based on the data from clinical cases, the BA.5 variant dominated, peaking at 99% in October. Subsequently, the BQ.1 variant became more prevalent, reaching its peak at 62% by the end of January 2023. By the end of February, the BA.2.75 variant started to increase in prevalence. In February 2023, the XBB variants with sublineages began to rise. In June, the BA.1 variant emerged, followed by a surge in the XBB1.5-like and additional spike mutation F456L from the end of October and the BA.2.86 variant. The abundance of SARS-CoV-2 variants by individual WWTP is displayed in Supplementary Figure S2.

## 4. Discussion

### 4.1. Overview of the Selected Sampling Sites

Slovenia has a population of 2,116,972 inhabitants [7] living in 212 municipalities, which are spread across 12 statistical regions. The country is known for its scattered and sparsely populated areas [20]. Nevertheless, each statistical region has at least one major city. For our sampling sites, we aimed to include representation from all 12 statistical regions, selecting the largest WWTPs in each region. This approach resulted in the inclusion of 16 major WWTPs in the monitoring system, covering a population of 730,887 Slovenian inhabitants (34.2% of the total population).

The findings from the pilot monitoring demonstrated that monitoring at the 16 WWTPs was sufficient for effectively tracking SARS-CoV-2 infections at both the national and regional levels. The coverage achieved in Slovenia is comparable to that of Sweden [21]. Although some other European countries have higher coverage rates, such as Finland with 44% [22], Belgium with 45% [23,24], and Austria with 58% [25], our system’s results were still substantial.

**Figure 7:**
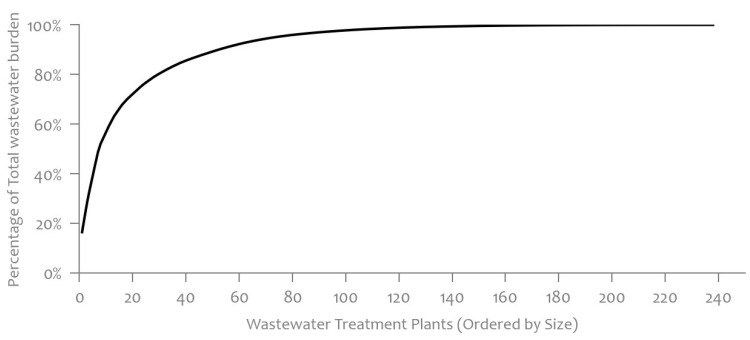
Distribution of total wastewater burden percentage by WWTPs size [9].

If we would aspire to attain even higher coverage rates in Slovenia, we would need to incorporate additional smaller WWTPs into the monitoring system. However, due to their smaller capacities, including these WWTPs would not significantly increase the population coverage. Figure 7 accompanying this analysis further supports this observation. It illustrates the distribution of wastewater burden among WWTPs, revealing that a significant portion of the total wastewater burden is managed by a limited number of larger WWTPs. Specifically, in Slovenia, there are 246 WWTPs with environmental permits, out of which 20 have a capacity of ≥20,000 population equivalent, and only 13 have a capacity of ≥50,000 population equivalent [8]. To achieve a significantly higher coverage rate, a large number of small WWTPs would have to be included, resulting in unproportionable higher costs compared to the added value.

Numerous methodologies for the detection and quantification of SARS-CoV-2 in wastewater have been described in the literature [26,27]. The workflow used in the present study underwent verification, establishing its reliability for generating stable and reproducible results. In addition to the endogenous control PMMoV, we included the EAV virus as recovery control for each sample. However, wastewater samples present a material with complex and variable composition, thus it is essential to have accurate and defined control parameters. In instances where control parameters were suboptimal, data were excluded. For instance, if PMMoV concentrations fell outside of the average concentrations ± standard deviation for specific WWTP.

Moreover, although an amplicon sequencing approach was employed to determine SARS-CoV-2 variants, insufficient sequencing data were obtained when virus concentrations were low (Ct values below 35). This limitation resulted in missing variant results for specific dates.

### 4.2. Estimated Number of SARS-CoV-2 Infections Per 100,000 Catchment Population

A wastewater-based indicator, the estimated number of SARS-CoV-2 infections per 100,000 catchment population, was utilized to predict the incidence rate that would have been observed in clinical data had the testing rate of human cases remained consistent prior to the reclassification of COVID-19 as an endemic respiratory disease on 1 April 2023. From that date on, the testing strategy has shifted from population screening to a more focused approach, by testing symptomatic persons, specifically persons hospitalized due to severe acute respiratory infections. The extent of testing rate reduction is also visible in Figure 2 [3]. Subsequently, wastewater-based indicators became a key source of information on the COVID-19 epidemic in the endemic period in Slovenia.

The observed trends in the incidence of both estimated SARS-CoV-2 infections and active clinically confirmed infections offer valuable insights into the dynamics of the COVID-19 epidemic in Slovenia in the observed surveillance period (September 2022 to December 2023).

The estimated infections ranged from a peak of 2312.3 per 100,000 catchment population in September 2022 to a low of 60.8 per 100,000 catchment population in June 2023. Notably, following an initial peak in September 2022, there was a gradual decline until January 2023, followed by a resurgence in February 2023, with subsequent peaks in March–April 2023 and December 2023. Importantly, the magnitudes of these peaks relative to the initial peak in September 2022 demonstrate a notable decrease over time. Similarly, the trends in active clinically confirmed infections, with a peak of 1026.8 per 100,000 inhabitants in October 2022 and a low of 1.1 per 100,000 inhabitants in July 2023, mirror the patterns observed in estimated infections. The initial peak in October 2022 was followed by a gradual decline until January 2023, after which a resurgence occurred, with peaks in April and June 2023, along with another peak in November 2023. In early May 2023, the estimated number of infections from wastewater monitoring began to decline. Since then, the estimated values have not shown significant fluctuations. A similar epidemic trajectory was also observed through wastewater monitoring in Austria [25] and Germany [28]. The reduction of estimated infections in May 2023 aligns with the seasonal pattern of SARS-CoV-2 infections observed in the clinical surveillance system in Slovenia between the years 2020 to 2022. The infections reached the lowest trend in the period between June and August 2023 [4].

Comparing the peaks in estimated and active clinically confirmed infections reveals interesting dynamics. While both indicators exhibited similar trends, the peaks in estimated infections were consistently higher. This discrepancy likely arises from undetected cases within the clinical surveillance system, such as asymptomatic individuals or those with mild symptoms who did not seek testing. This gap became more evident after COVID-19 was declared endemic, along with changes in testing strategies, which further limited the detection of infections through clinical testing. Similar findings were observed in studies [29,30] where wastewater data captured broader infection dynamics, highlighting the underreporting in clinical testing.

The validation of the wastewater indicator estimated number of SARS-CoV-2 infections per 100,000 catchment population using cross-correlation analysis revealed promising results regarding the association between wastewater indicator and active clinically confirmed infections. The best fit between data was observed at a lag of 1 week. Out of the 16 WWTPs, all exhibited statistically significant correlations. Specifically, five WWTPs demonstrated strong correlations, while the remaining displayed moderate correlations. These findings suggest a varying degree of alignment between the two surveillance methods across different geographic locations within Slovenia. The variability in correlation strength between wastewater indicators and clinical data across different WWTPs and their regions suggests that several regional factors could influence the effectiveness of wastewater-based surveillance. These factors may include differences in population density, catchment area size, wastewater flow rates, and the proportion of asymptomatic cases in the population. Additionally, the efficiency of viral shedding in wastewater, influenced by factors such as weather conditions, sewer infrastructure, and delays in wastewater collection, may contribute to the observed variability. Similar factors were considered in study [31].

In WWTPs like Ljubljana, Postojna, Domžale-Kamnik, Kranj, and Šaleške doline, the strong correlations observed may be a result of more consistent wastewater flow rates relative to population size, as well as higher case detection rates in clinical settings. On the other hand, moderate correlations at other WWTPs may reflect more dispersed populations, potential delays in clinical reporting, or variability in virus-shedding patterns. To further explore these differences, it would be beneficial to analyze WWTPs’ characteristics (e.g., sources of wastewater, primary vs. secondary treatment) and evaluate regional healthcare accessibility, which may influence both wastewater surveillance data and clinical case detection.

### 4.3. Abundance of Mutations Specific to SARS-CoV-2 Variants

By comparing the results of the sequencing of wastewater samples to the results from the Slovenian clinical case surveillance system, as displayed in Figure 4 and Figure 5, we can obtain the following findings. Initially, starting from September 2022, both wastewater surveillance and clinical case data identified the predominance of the BA.5 variant. However, discrepancies emerged as the study progressed. While wastewater analysis indicated a gradual decrease in BA.5 dominance from November 2022, clinical data continued to report its prevalence until early December 2022. Furthermore, the emergence of the BQ.1 variant, initially found in lower abundances in wastewater samples, gained significance in clinical cases, reaching a peak of 62% by the end of January 2023. The divergence in variant prevalence between the two systems became more pronounced in early 2023. Clinical data highlighted the spread of the BA.2.75 variant alongside the emergence of XBB variants, while wastewater analysis primarily identified the prevalent virus variants belonging to the broader Omicron parent lineage. Notably, surveillance of clinical cases detected a small prevalence of BA.1 and BA.2 variants not identified in wastewater samples. From June 2023 onwards until the first half of December 2023, XBB variants with sublineages dominated wastewater samples. Towards the end of 2023, a notable prevalence of EG.5, the predominant lineage within the XBB.1.5-like variant carrying the F456L spike mutation, was observed in wastewater samples. Concurrently, the presence of the BA.2.86 variant was also noted during this period.

These discrepancies underscore the importance of integration of multiple data sources for a comprehensive understanding of SARS-CoV-2 variant dynamics. Wastewater surveillance, implemented consistently throughout the entire study period, covering various Slovenian regions, provided a broad picture of variant prevalence. However, its efficacy was hindered during periods of low virus prevalence, such as the summer months. At that time, the identification of virus variants was limited to the level of Omicron parent lineage. On the other hand, clinical surveillance faced its own limitations. This included a significant drop in testing rates after February 2023, with the sequencing strategy primarily focused on severe cases. Moreover, the percentage of sequenced clinical samples in the period before the significant drop in testing rate, as observed in Figure 2, was below 5%, raising questions about its representativeness [4]. Additionally, longer turnaround times for sequencing results compared to wastewater analysis further hindered the timeliness of clinical surveillance data for preparedness and response.

These insights collectively offer a comprehensive picture of the SARS-CoV-2 variant dynamics in the community, with wastewater surveillance providing population-wide trends and clinical testing pinpointing specific variants in diagnosed cases [4].

### 4.4. Significance of Wastewater Surveillance System in the Endemic Phase of the SARS-CoV-2 Disease

With the transition of COVID-19 into an endemic form of the disease, the needs and expectations for its monitoring have changed. Effective public health response now requires monitoring the trends in virus circulation within the population, the detection of outbreaks, and tracking the variants emerging in the population. This enables the timely detection of potential increases in infections and the emergence of new variants that could lead to altered epidemiological situations. In the context of pandemic preparedness, the data collected through wastewater surveillance is crucial for proactive measures, contributing to effective disease control and management [32]. Monitoring SARS-CoV-2 in wastewater is a suitable tool for tracking these trends, as it provides an earlier indication of trend changes compared to monitoring human testing and allows for quicker responses [33,34,35]. In addition, monitoring infected individuals with SARS-CoV-2 through wastewater includes all individuals connected to a specific WWTP, regardless of whether they have noticeable symptoms, including those with mild symptoms or without symptoms [36].

After the pilot phase, the monitoring of SARS-CoV-2 in wastewater, presented in the form of a dashboard, has been implemented on the National Institute of Public Health website, which facilitates effective communication with the general public, professionals, and decision-makers. Additionally, the weekly public health risk assessment report from the Emergency Operations Centre, which included wastewater trends, was submitted to the government to help shape its measures, and to hospitals so they could anticipate and adjust their capacity needs. With this report over 358 healthcare personnel from 64 healthcare facilities, 15 general hospitals, 5 psychiatric hospitals, 130 nursing homes, and 24 pharmacies were reached [37,38].

### 4.5. Limitations and Advantages

Wastewater surveillance systems pose several challenges [32,34,39]. They can generate false-positive results due to high sensitivity when poor RT-PCR assays are used [26]. In areas with inadequate sanitation and limited WWTPs, the effectiveness of this surveillance may be compromised [40]. Challenges arise in linking identified infected cases to virus levels because of variations in viral excretion, different periods, population movement, and dilution from precipitation [41]. Genome stability risks in wastewater, variations in sampling techniques, inefficient viral concentration, and the lack of sensitive screening assays can limit viral detection, especially at low concentrations. Additionally, emerging diagnostic methods may lack universal healthcare approval. Complex stakeholder involvement and the need for precise data calibration further complicate the process [32].

An additional limitation observed in our study is the low population-to-flow ratio across the selected WWTPs (0.002–0.006). Previous research has demonstrated that low population-to-flow ratios weaken the correlation between SARS-CoV-2 RNA concentrations in wastewater and COVID-19 case counts, as the dilution effect can make viral signals less detectable [31]. This dilution likely played a role in limiting the sensitivity of our surveillance system, making it more difficult to accurately correlate wastewater viral loads with infection trends in the monitored regions.

While this study provides valuable insights into the correlation between wastewater surveillance data and clinically confirmed COVID-19 cases, we acknowledge that additional statistical analyses, such as significance tests and multivariate models, were not applied. These analyses could help further assess site-specific differences and temporal trends, offering a more robust understanding of the surveillance system’s effectiveness in different geographical contexts. Future work will focus on incorporating these methods to enhance the sensitivity and specificity of our surveillance approach, not only for SARS-CoV-2 but also for other pathogens, as our system is still under development.

These limitations underscore the importance of continuous research and refinement to enhance wastewater surveillance’s accuracy and effectiveness. Our surveillance system shares many of the challenges observed in other countries, including variations in viral excretion rates, dilution effects due to low population-to-flow ratios, and differences in sampling techniques. To address these challenges, the EU Commission’s “Cookbook” [42] has recently provided a framework for standardizing wastewater surveillance methodologies across Europe, aimed at improving accuracy and comparability of results. As Slovenia continues to refine its system, incorporating these harmonized approaches will be essential for increasing both coverage and accuracy, particularly for surveillance of pathogens beyond SARS-CoV-2.

## 5. Conclusions

Monitoring SARS-CoV-2 in wastewater detected seasonal surges of infections in the winter 2022 and the spring 2023 decline in a timely manner, contributing to the stabilization of the epidemiological situation. The winter increase was associated with the spread of new SARS-CoV-2 variants, which were promptly identified through wastewater surveillance. As human testing gradually decreased, the role of monitoring SARS-CoV-2 in wastewater became increasingly important. Today, in Slovenia, the wastewater monitoring of SARS-CoV-2 is the most important method that allows for tracking trends in virus spread within the population and the only method that allows tracking SARS-CoV-2 variants within the population. This provides the capability for early detection of new variants.

## Figures and Tables

**Table 1:** Validation results using cross-correlation at lag 1 (1 week).

Wastewater Treatment Plant	Correlation Coefficient	*p*-Value	Strength of Correlation
Brežice	0.41	<0.05	Moderate
Litija in Šmartno pri Litiji	0.50	<0.05	Moderate
Ljubljana	0.72	<0.05	Strong
Murska Sobota	0.43	<0.05	Moderate
Postojna	0.82	<0.05	Strong
Slovenj Gradec	0.51	<0.05	Moderate
Trbovlje	0.57	<0.05	Moderate
Zagorje	0.47	<0.05	Moderate
Celje	0.42	<0.05	Moderate
Domžale-Kamnik	0.74	<0.05	Strong
Koper	0.55	<0.05	Moderate
Kranj	0.73	<0.05	Strong
Maribor	0.66	<0.05	Moderate
Nova Gorica	0.59	<0.05	Moderate
Novo mesto	0.48	<0.05	Moderate
Šaleške doline	0.70	<0.05	Strong

## Data Availability

Data supporting the findings of this study are available from the corresponding author upon reasonable request.

## Supplementary Materials

The following supporting information can be downloaded at: https://www.mdpi.com/article/10.3390/microorganisms12112174/s1, Table S1: Monitored Wastewater Treatment Plants, Municipalities, and Population Coverage by Statistical Region; Table S2: Primers and probes used in laboratory analysis; Table S3: Standard curve parameters and limit of detection for qPCRs; Figure S1: Time-series plot of the estimated number of SARS-CoV-2 infections per 100,000 inhabitants for individual WTTPs and number of active clinically confirmed SARS-CoV-2 infections per 100,000 people in the catchment population, September 2022–December 2023; Figure S2: Abundance of SARS-CoV-2 variants, as determined based on the wastewater data, at individual WWTPs, September 2022–December 2023. References [43,44] are cited in the Supplementary Materials.
