# Supplementary material for: Wastewater Surveillance of SARS-CoV-2 in Slovenia: Key Public Health Tool in Endemic Time of COVID-19
Source: Microorganisms. 2024 Oct 29;12(11):2174. doi: 10.3390/microorganisms12112174 (PMC11596113; doi:10.3390/microorganisms12112174)
Supplement: Supplementary file 1 [file microorganisms-12-02174-s001.zip › microorganisms-3256312-supplementary.pdf]

Supplementary data - Tables.

Table S1: Monitored Wastewater Treatment Plants, Municipalities, and Population Coverage by Statistical Region.

Statistical Region	Wastewater Treatment Plants	Municipalities	Population Coverage*
Central Slovenia	Ljubljana, Domžale-Kamnik	Ljubljana, Kamnik, Domžale, Mengeš, Trzin, Komenda	53.6%
Upper Carniola	Kranj, Domžale-Kamnik	Cerklje na Gorenjskem, Kranj, Naklo, Šenčur	26.9%
Savinja	Celje, Šaleške doline	Celje, Štore, Velenje, Šoštanj	29.0%
Coastal-Karst	Koper	Koper, Izola, Ankaran	45.5%
Drava	Maribor	Maribor, Miklavž na Dravskem polju, Duplek, Hoče-Slivnica	33.5%
Gorizia	Nova Gorica	Nova Gorica, Brda, Šempeter-Vrtojba, Miren-Kostanjevica	21.5%
Central Sava	Trbovlje, Litija in Šmartno pri Litiji, Zagorje	Trbovlje, Litija, Šmartno pri Litiji, Zagorje ob Savi	54.2%
Littoral-Inner Carniola	Postojna	Postojna	17.1%
Sautheast Slovenia	Novo mesto	Novo mesto	16.7%
Lower Sava	Brežice	Brežice	10.2%
Carinthia	Slovenj Gradec	Slovenj Gradec	17.2%
Mura	Murska Sobota	Murska Sobota	15.2%

* The proportion of the statistical region's population included within the Wastewater Treatment Plants catchment population.

Table S2: Primers and probes used in laboratory analysis.

Primers and probes	Sequence (5' – 3')	References
Covid-N3-F	ggg AgC CTT gAA TAC ACC AAA Ag	(Yaniv *et al.* 2021)
Covid-N3-R	TgT AgC ACg ATT gCA gCA TTg	
Covid-N3-S	6FAM – TCA CAT Tgg CAC CCg CAA TCC TgC – BBQ	
PMMoV-F	gAgTggTTTgACCTTAACgTTTgA	(Kitajima *et al*. 2018)
PMMoV-R	TTgTCggTTgCAATgCAAgT	
PMMoV-S	Cy5-CCTACCg+A+Ag+CAAATg—BBQ	

Table S3: Standard curve parameters and limit of detection for qPCRs.

Target	Range (GC/reaction)	Standard curve parameters	LOD (GC/reaction)
N3	101-106	R^2: 0.996; E: 96.3%; intercept: 40.743; slope: -3.41	10
PMMoV	101-106	R^2: 0.990; E: 96.3%; intercept: 41.666; slope: -3.41	10
EAV	101-106	R^2: 0.999; E: 101.6%; intercept: 38.80; slope: -3.28	10

Supplementary data - Figures.

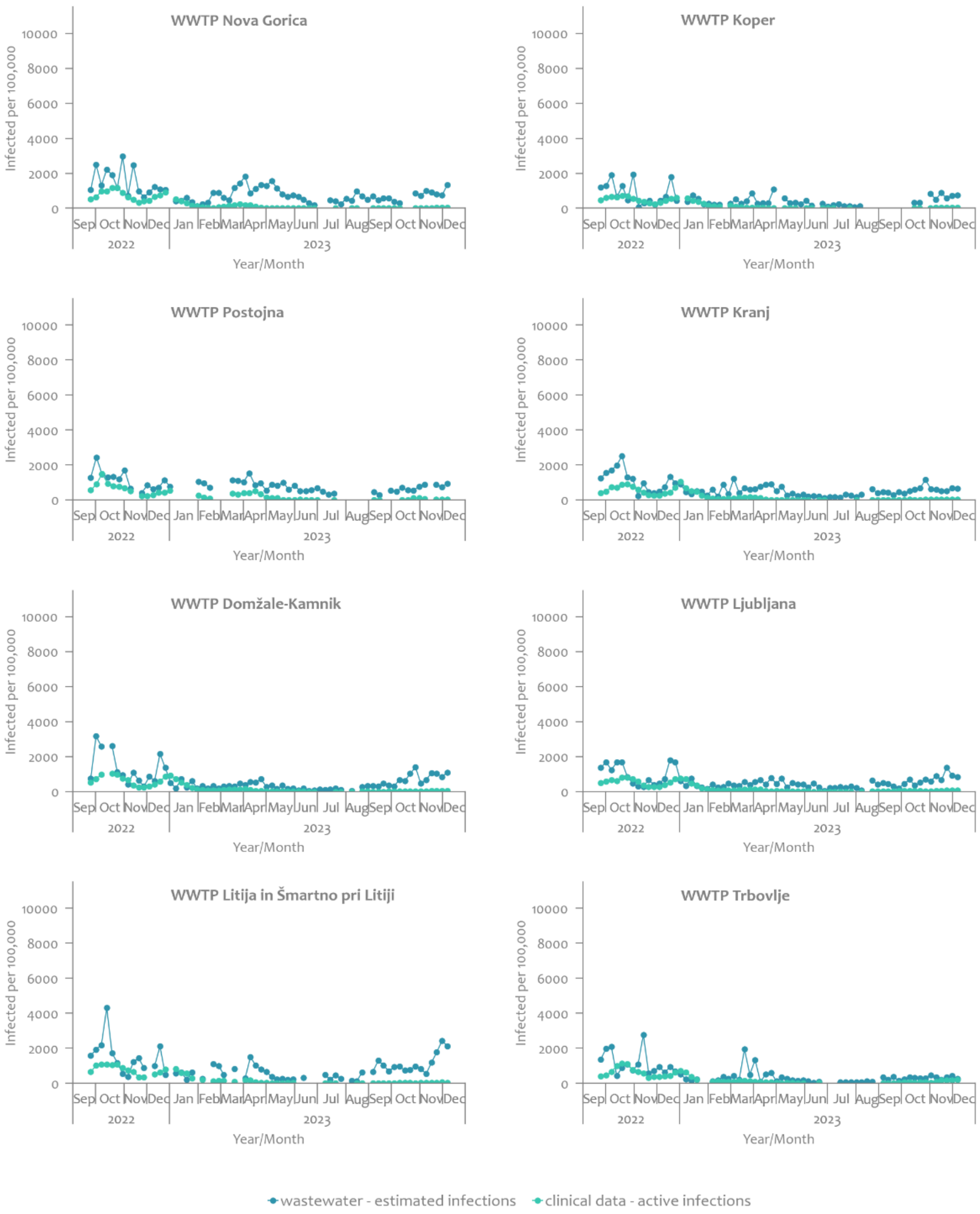

Figure S1: Time-series plot of the estimated number of SARS-CoV-2 infections per 100,000 inhabitants for individual WTTPs and number of active clinically confirmed SARS-CoV-2 infections per 100,000 people in the catchment population, September 2022 – December 2023.

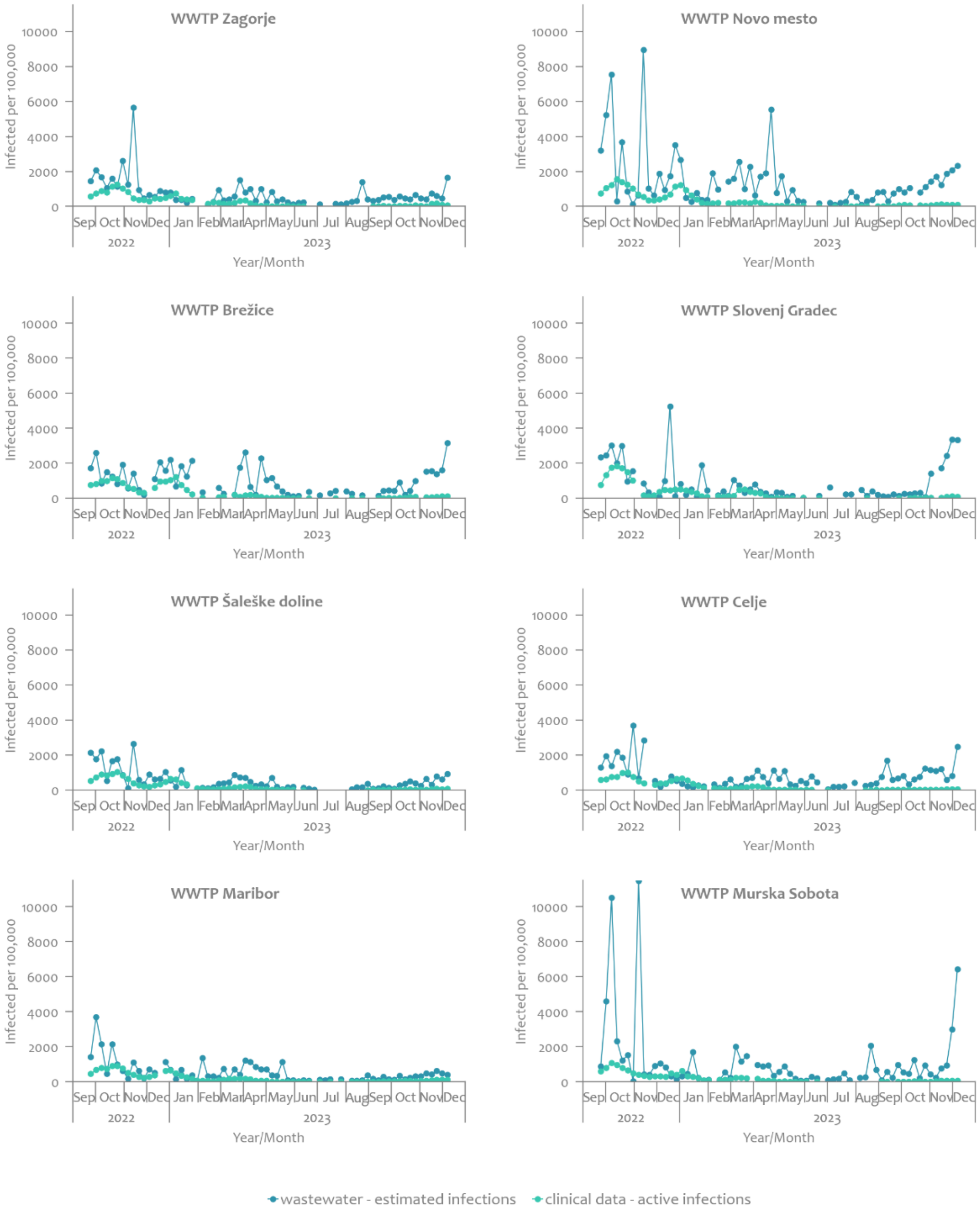

Figure S1 (continued): Time-series plot of the estimated number of SARS-CoV-2 infections per 100,000 inhabitants for individual WTTPs and number of active clinically confirmed SARS-CoV-2 infections per 100,000 people in the catchment population, September 2022 – December 2023.

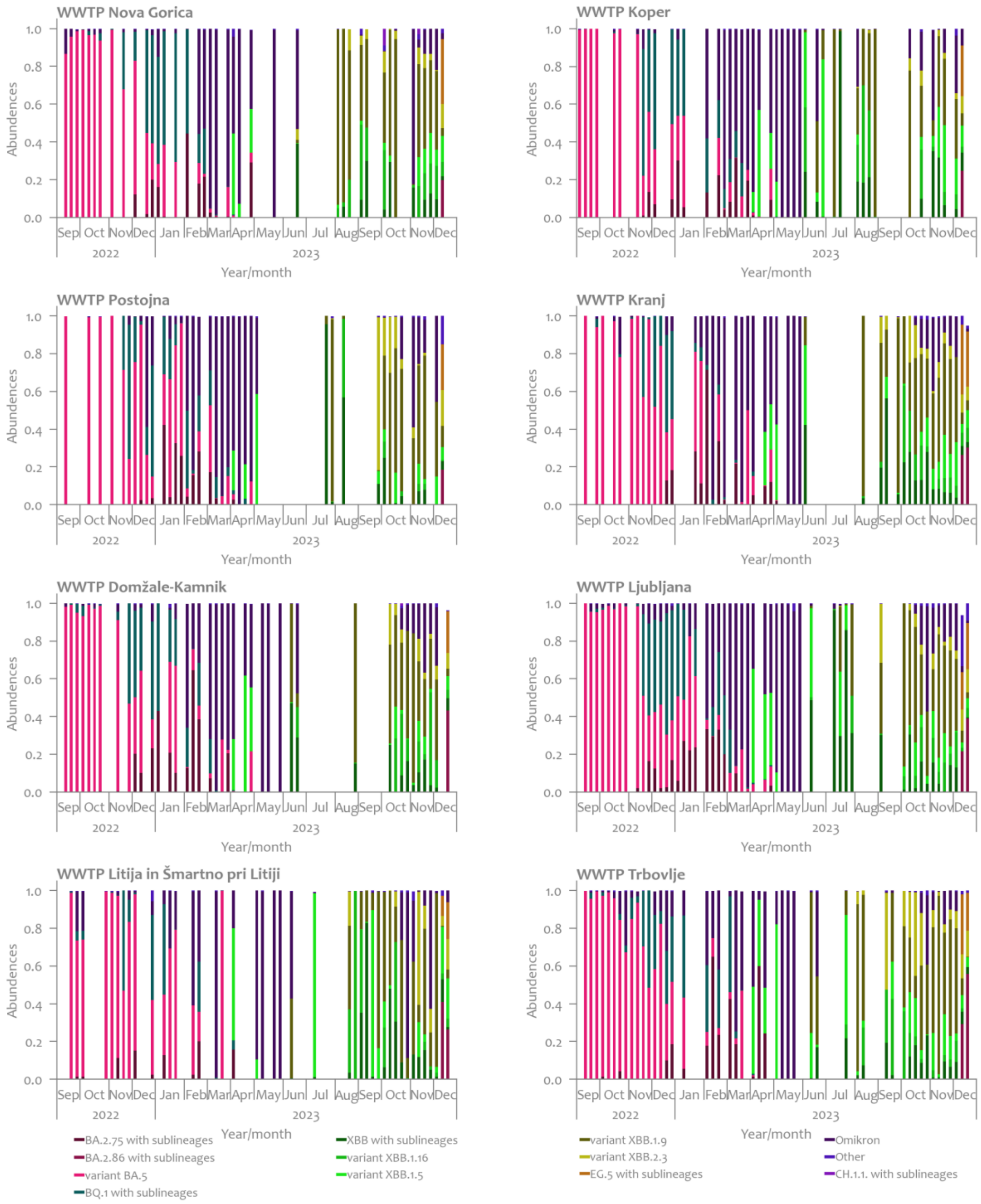

Figure S2: Abundance of SARS-CoV-2 variants, as determined based on the wastewater data, at individual WWTPs, September 2022 – December 2023.

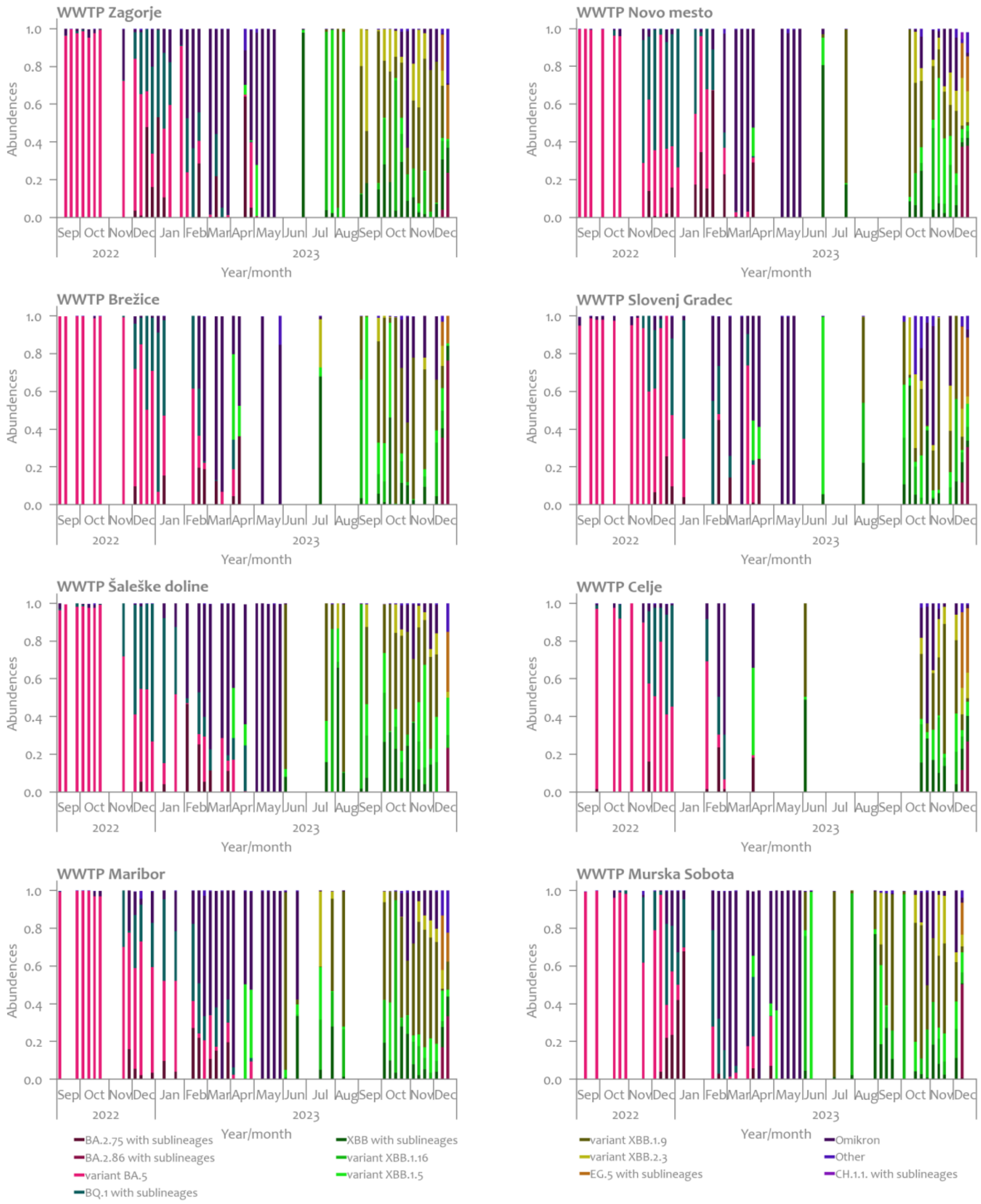

Figure S2 (continued): Abundance of SARS-CoV-2 variants, as determined based on the wastewater data, at individual WWTPs, September 2022 – December 2023.
